# Studying the Interactions of U24 from HHV-6 in Order to Further Elucidate Its Potential Role in MS

**DOI:** 10.3390/v14112384

**Published:** 2022-10-28

**Authors:** Keng-Shuo Pi, Daria Bortolotti, Yurou Sang, Giovanna Schiuma, Silvia Beltrami, Sabrina Rizzo, Alessandra Bortoluzzi, Eleonora Baldi, A. Louise Creagh, Charles A. Haynes, Roberta Rizzo, Suzana K. Straus

**Affiliations:** 1 Department of Chemistry, University of British Columbia, 2036 Main Mall, Vancouver, BC V6T 1Z1, Canada; 2 Department of Chemical, Pharmaceutical and Agricultural Sciences, University of Ferrara, 44121 Ferrara, Italy; 3 Faculty of Health Sciences, Simon Fraser University, 8888 University Drive, Burnaby, BC V5A 1S6, Canada; 4 Rheumatology Unit, Department of Medical Sciences, University of Ferrara and Azienda Ospedaliero-Universitaria S. Anna, 44121 Ferrara, Italy; 5 Department of Neuroscience and Rehabilitation, Division of Neurology, “Sant’Anna” University-Hospital, 44121 Ferrara, Italy; 6 Michael Smith Laboratories and Department of Chemical and Biological Engineering, University of British Columbia, 2185 East Mall, Vancouver, BC V6T 1Z4, Canada

**Keywords:** human herpes virus, *Roseolovirus*, Fyn-SH3, Nedd4L, multiple sclerosis, phosphorylation, natural killer cells, Killer Cell Immunoglobulin Like Receptor 2DL2

## Abstract

A number of studies have suggested that human herpesvirus 6A (HHV-6A) may play a role in multiple sclerosis (MS). Three possible hypotheses have been investigated: (1) U24 from HHV-6A (U24-6A) mimics myelin basic protein (MBP) through analogous phosphorylation and interaction with Fyn-SH3; (2) U24-6A affects endocytic recycling by binding human neural precursor cell (NPC) expressed developmentally down-regulated protein 4-like WW3* domain (hNedd4L-WW3*); and (3) MS patients who express Killer Cell Immunoglobulin Like Receptor 2DL2 (KIR2DL2) on natural killer (NK) cells are more susceptible to HHV-6 infection. In this contribution, we examined the validity of these propositions by investigating the interactions of U24 from HHV-6B (U24-6B), a variant less commonly linked to MS, with Fyn-SH3 and hNedd4L-WW3* using heteronuclear single quantum coherence (HSQC) nuclear magnetic resonance (NMR) titrations and isothermal titration calorimetry (ITC). In addition, the importance of phosphorylation and the specific role of U24 in NK cell activation in MS patients were examined. Overall, the findings allowed us to shed light into the models linking HHV-6 to MS and the involvement of U24.

## 1. Introduction

Human herpesviruses HHV-6A, -6B and -7 are *Roseoloviruses* that have been hypothesized to be implicated in several neurological diseases, including encephalitis, epilepsy, Alzheimer’s and multiple sclerosis (MS) [1,2,3,4,5,6,7]. Although the exact cause of MS is still currently unknown, it has been suggested that one or more viruses may be possible triggers [8,9,10,11,12]. Specifically, it has been proposed that potential triggering agents, such as Epstein–Barr virus, HHV-6 (the collective name for HHV-6A and HHV-6B), varicella–zoster virus, cytomegalovirus, C virus and human endogenous retroviruses, play a role [11,13,14]. A number of these viruses are ubiquitous and highly prevalent in adult populations worldwide [15]. To date, only a limited number of potential viral proteins that could be explicitly involved in this triggering have been identified [16,17,18,19,20,21,22,23].

U24 (see Supplemental for nomenclature), a putative tail-anchored membrane protein that is unique to *Roseoloviruses* [24], is one such protein that may be implicated in MS. It is expressed during the early stages of viral infection [25]. Although the exact function of U24 has not been fully elucidated, a number of studies have suggested a potential link between U24 and MS [15,26,27,28,29,30,31]. In HHV-6A, the N-terminal segment of U24 consists of a PxxP motif, as well as a PY motif (Table 1). These same two motifs can be found in U24 from HHV-6B, which is not surprising given that HHV-6A and -6B genomes share an overall identity of 90%. Finally, in HHV-7, U24 only possesses the PY motif (Table 1). The proline-rich segment of U24 has been suggested as being important for its function, as such segments are typically involved in protein–protein interactions because of the distinct structure of the polyproline helix they form [13]. A number of important protein–protein interactions involving prolines and linked to MS will be described below.

Both U24-6A and -6B share a proline rich segment with myelin basic protein (MBP, Table 1), a key component of the myelin sheath protecting axons in the central nervous system (CNS). In MS, MBP becomes an autoantigen, i.e., a normal constituent of neuronal cells that becomes the target of an immune response from auto-reactive T-cells [9,10,32]. The seven amino acid sequence identity between U24 from HHV-6 and MBP has led to the suggestion that U24 may function by mimicking MBP. Indeed the polyproline region in MBP is essential for it to participate in Fyn-mediated signaling pathways via a direct but non-canonical association between the Fyn-SH3 domain and the PxxP motif [33]. Oligodendrocyte differentiation and maturation, which are processes that are perturbed in MS patients, are impacted by the disruption of these signaling pathways. In addition, MBP has two threonine residues (T95 and T98) within and just before the PxxP motif (Table 1), which can be phosphorylated. Phosphorylation has been shown to affect the local structure of MBP and its disposition on the membrane surface [34]. In addition, MS patients have a degree of phosphorylation at T98 that is lower than that of normal individuals [35]. Phosphorylation was found to decrease the ability of MBP to polymerize actin and to bundle actin filaments, without however affecting the dissociation constant of the MBP-actin complex [36]. Additionally, phosphorylation of MBP leads to a reduced interaction with Fyn-SH3 [37]. Finally, MBP phosphorylation decreases the interaction between MBP and lipids, making it difficult to organize lipids into the multilayers found in the myelin sheath [38]. U24 from HHV-6A has been shown to associate with Fyn-SH3, albeit ca. 1000-fold less than MBP (*K_d_* = 5 mM for U24-6A [29] versus 4-8 μM for MBP [39]). In addition, U24-6A can be phosphorylated by MAPK, but less effectively than MBP [28]. Overall, these findings suggested that although U24-6A may mimic MBP, it most likely does not interfere directly with MBP function.

U24 has been shown to mediate the downregulation of the T-cell receptor complex and also the transferrin receptor (TfR) through its PY motif [40,41], suggesting a general block in early endosomal recycling. These results strongly suggest that U24 expression can influence the activation state of the immune system of infected individuals, supporting the potential link between U24 and MS immune dysregulation [15,26,27,28,29,30,31]. Endosomal recycling is a process controlled by E3 ubiquitin ligases. Recently, U24-6A and the phosphorylated pU24-6A have been found to preferentially interact with neural precursor cell (NPC) expressed developmentally down-regulated protein 4 (Nedd4) E3 ubiquitin ligase, via its WW3 domains [30,31]. In the CNS, Nedd4 and Nedd4-like proteins (Nedd4L) play a crucial role in promoting dendrite outgrowth [42,43] and maintaining neuronal survival [44,45]. So hypothetically, U24 could function by affecting Nedd4 or Nedd4L, which may cause defective neural development, resulting in damage of axons or myelin.

On the other hand, U24 may play a role in HHV-6 infectivity. Studies have demonstrated that MS patients who express Killer Cell Immunoglobulin Like Receptor 2DL2 (KIR2DL2) on natural killer (NK) cells are more susceptible to HHV-6 infection [46,47,48]. In particular, Rizzo et al. have shown reduced NK cell activation and consequently a low HHV-6 clearance in MS patients with a KIR2DL2 allele. KIRs are MHC class I-specific regulatory receptors utilized by human NK cells and CD8 T cells. Several lines of evidence link differences in KIR expression to differential responses to invading pathogens and autoimmune disorders [49,50,51,52]. To date, no studies have demonstrated whether U24 plays a direct role in NK cell activation in MS patients.

Given previous findings on U24-6A and U24-7 [29,30,31] and in order to better understand the potential role of U24 in MS and the features of U24 that are important for the protein–protein interactions discussed above, we have examined here the interaction of U24 from HHV-6B and its phosphorylated form with hNedd4L-WW3* (3rd WW domain in human Nedd4L, where the star was adopted in literature describing sequence comparisons of the human vs. rat/mouse analogues) and Fyn-SH3. Since HHV-6A is more commonly linked with MS than HHV-6B [27,53,54,55], it is hypothesized that U24-6B binding to hNedd4L-WW3* might be weaker than for U24-6A. In addition, a number of studies have shown that the interaction between proline-rich segments and Fyn-SH3 is highly dependent on the presence of arginine residues that flank the PxxP motif [56,57,58,59,60]. Hence, it is anticipated that U24-6B, with an Arg at position 3 (Table 1) and an Asp at position 2, might bind more tightly to Fyn-SH3 than U24-6A. Finally, since NK cell recognition and specific killing of HHV-infected cells are tightly regulated by KIRs and their MHC-ligands, it would be important to establish the role of KIRs in MS with a particular attention to demonstrating preferential natural killer (NK) cell activation in MS patients upon exposure to U24-6A and -6B and their respective phosphorylated versions.

## 2. Materials and Methods

### 2.1. Ethics Statement

The part of this study involving human subjects was conducted following ethics approval by the Area Vasta Emilia Centro (N:01052016), who also approved the experimental protocols used. The study adheres to the ethical principles for medical research involving human subjects as required by the 2013 revision of the Declaration of Helsinki–WMA Declaration of Helsinki–Ethical Principles for Medical Research Involving Human Subjects. Furthermore, none of the female MS patients, Neurolupus (NLES) and control subjects were pregnant before entering the study.

### 2.2. Synthesis and Purification of 15-Residue Peptides Representing the Polyproline-Rich N-Terminal Region of U24 from HHV-6A and HHV-6B

Two 15-mer peptides representing the N-terminal region of U24-6A and U24-6B (Table 1) were synthesized using N-9-fluorenylmethyloxycarbonyl (N-Fmoc) protected α-amino acids on an automated peptide synthesizer (CS Bio, Menlo Park, CA, USA). Preloaded Fmoc-Leu-Wang resin (Advanced ChemTech, Louisville, KY, USA) and O-(benzotriazol-1-yl)-N,N,N′,N′-tetramethyluronium hexafluorophosphate (HBTU, Advanced ChemTech) were used as the coupling reagents. Double coupling was required for the last nine residues of U24-6A (MDPPRTPPP) and U24-6B (MDRPRTPPP). The crude peptide was deprotected and cleaved from the resin in 90% trifluoroacetic acid (TFA) with trace ddH_2_O, 1,2-ethanedithiol (EDT) and triethylsilane (TES) as scavengers for at least 5 h. The excess organic solvents were removed under reduced pressure, and the peptide was precipitated through careful addition of 100 mL of cold diethyl ether into the peptide mixture. The filtered crude peptide powder was then dried under vacuum in a desiccator overnight, redissolved and frozen in de-ionized water and lyophilized to remove all residual solvents. Then, the presence of the desired peptide was determined by MALDI-TOF MS.

Both U24-6A and U24-6B peptides were purified using a C18 reverse-phase high-performance liquid chromatography (HPLC) column (Phenomenex, Torrance, CA, USA, Jupiter, 10 μm 300 Å, 250 × 21.2 mm) on a Waters 600 system, monitored by UV absorption at 228 nm and 278 nm using a photodiode array detector. U24-6A peptide was eluted with a gradient from 0 to 15% of acetonitrile for over 28 min at a flow rate of 10 mL/min. In contrast, U24-6B peptide was eluted with a gradient from 0 to 30% of acetonitrile for over 50 min at a flow rate of 10 mL/min. The purified peptides were lyophilized and HPLC purification procedure was repeated a total of four times to ensure high purity (>=95%, Figure S1).

Phosphorylated peptides (with a phospho-threonine 6, Table 1) were purchased from Genscript (Piscataway, NJ, USA) and used without further purification.

### 2.3. Expression and Purification of hNedd4L-WW3*

The pGEX-4T2 vector plasmid used here was previously described by Sang et al. [30]. The plasmid was transformed into E. coli BL21(DE3) for expression according to standard protocols. In preparation for large-scale expression, 5 mL of starting Luria Bertani- carbenicillin (LB-CBC) culture was firstly grown (37 °C; 5 to 6 h; 225 rpm). Then, 1 mL of the starting culture was used to inoculate 800 mL of fresh LB-ampicillin (amp). The large culture was then grown (37 °C; 5 to 6 h; 225 rpm) till OD_600_ reached 0.5, and cooled down in the cold room for 5 min before induction (16 h; 25 °C; 225 rpm) with 400 μM IPTG. 500 μL samples from before and after induction were pelleted via centrifugation for SDS-PAGE analysis to confirm protein expression. The cells were harvested via centrifugation (5000 rpm; 20 min; 4 °C) and the remaining cell paste from each 800 mL culture was washed once in 30 mL PBS buffer, centrifuged (3900 rpm; 10 min; 4 °C) and stored in −80 °C.

The cell paste was thawed on ice and resuspended initially in 5 mL of PBS/Triton buffer (PBS buffer supplemented with 1% Triton X-100). 10 mg of lysozyme, 20 μL protease inhibitor and 20 μL of DNAse were added to the mixture. The mixture was incubated on ice for 30 min, after which 5 mL of PBS/Triton buffer was added for a total volume of 10 mL. The mixture was then sonicated (2-s on-pulses; 5 s off-pulses; 37% amplitude power) in an ice bath for 3 min. After centrifugation (9000 rpm; 1 h; 4 °C), the supernatant was filtered using a 0.45 μm filter and then applied to 2 mL of PBS buffer washed Glutathione Sepharose 4B resin (GE Healthcare, Burnaby, BC, Canada, GST 4B resin), loaded into a 15 mL centrifuge tube. An end-over-end rotator (SARSTEDT) was used to mix the resin for 2 h at room temperature. Then, the 4B resin was washed with PBS/Triton buffer three times each, followed by sedimentation via centrifugation (3900 rpm; 5 min; 4 °C). Subsequently, the resin mixture was washed with Wash4 buffer once (0.5 mM Glutathione reduced in PBS buffer) and PBS buffer three times before adding 3 mL of 50 U/mL thrombin in PBS buffer to the 4B resin for an incubation period of 16 h at room temperature.

The supernatant containing cleaved WW3* domain (sequence in Table S1) was collected via centrifugation (3900 rpm; 10 min; 4 °C). The resin was washed with 4 mL of PBS buffer 2 more times before adding the cleaved solution into 200 μL of p-Amino benzamidine-agarose (Sigma-Aldrich, St.-Louis, MO, USA), washed with PBS buffer. The resin mixture was incubated at 4 °C for 2 h and pelleted by centrifugation (3900 rpm; 10 min; 4 °C). Then, the supernatant was washed with 100 μL of PBS washed fresh GST 4B resin to remove any residual GST protein in the cleaved WW3* domain fractions. 10 μL of the homogenate, supernatant, cell pellet, flow-through, thrombin cleavage product, thrombin wash product, and GST-wash product were collected and analyzed by Tris-Tricine SDS-PAGE with their concentrations adjusted accordingly. MALDI-MS was also used to confirm that the sequence listed in Table S1 was obtained.

### 2.4. Expression and Purification of ^15^N-Fyn-SH3

The plasmid used for the expression of GST-Fyn-SH3 protein was a kind gift from C. Pallen (Child & Family Research Institute, Vancouver, BC, Canada). The vector is pGEX kg with the SH3 domain of human Fyn (from residues TGVTLF to YVAPVD; Table S1) cloned into the HindIII–XbaI site [29]. The plasmid was transformed into E. coli BL21(DE3) for expression according to standard protocols.

In preparation for large-scale protein expression, 5 mL of starting LB-CBC culture was firstly grown (37 °C; 5 to 6 h; 225 rpm). A cell pellet was obtained via centrifugation (4 °C; 10 min; 3900 rpm) and removal of supernatant. The resultant cell pellet was dissolved and inoculated in 100 mL of ^15^N-M9-amp media supplemented with 1 g/l ^15^N-NH_4_Cl for a period of ca. 36 h (27 °C; 225 rpm). The 100 mL starting culture was then inoculated into 800 mL of ^15^N-M9-amp media for about 2 h, 37 °C and 225 rpm until OD_600_ reached 0.5–0.8 and cooled down in the cold room for 5 min before induction (4 h; 37 °C; 225 rpm), using 500 μM IPTG. 500 μL samples from before and after induction were pelleted via centrifugation for electrophoresis analysis to confirm protein expression. The cells were harvested via centrifugation (9000 rpm; 20 min; 4 °C) and the cell paste from each 800 mL culture was harvested as described in the preceding section.

Cleavage and purification were carried out using the identical protocols as described above for the purification of the WW domain, with the exception of one modification in the sonication procedure in which 5 s on-pulses and 10 sec off-pulses for 3 min were used instead. The purity of the obtained protein was verified using SDS-PAGE and MALDI-MS.

### 2.5. Subjects

40 MS Relapsing Remitting patients, in remission phase (mean age: 38 ± 10 years), 40 healthy controls (mean age: 37 ± 11 years) and 40 subjects with other inflammatory neurological diseases such as NLES (mean age: 36 ± 13 years) were recruited. MS was defined according to the classification of McDonald [61] and the patients were followed at the MS Centre of the Department of Neurology, University of Ferrara, Italy, during the period from 2015 to 2018. Disease disability was assessed in all MS patients at the time of sample collection using Kurtzke’s Expanded Disability Status Scale (EDSS) [62] (mean at entry: 2 ± 1, range from 0 to 5.5). All patients had Relapsing-Remitting course in agreement with the criteria of Lublin [63]. At the time of enrolment in the study, all the MS patients were clinically stable. At entry none of the patients had fever or other symptoms or signs of acute infections. Moreover, none of the patients had received any potential disease-modifying therapies (e.g., azathioprine or methylprednisolone, interferon-beta or glatiramer acetate) during the 6 months preceding the study. NLES patients satisfying the 1997 revised American College of Rheumatology criteria regularly attending the Lupus Clinic of the Rheumatology Unit, Department of Medical Sciences, Sant’Anna Hospital, University of Ferrara, Italy were recruited during the same period [64]. We recorded clinical, demographic, and serological data, as well as data regarding therapy, including corticosteroids (measured as prednisone equivalent), antimalarials, and immunosuppressants. Neuropsychiatric (NP) manifestations were defined according to the 1999 ACR nomenclature and case definitions and diagnostic work-up was performed according to the EULAR recommendations [65]. Attribution of NP events was based on physician judgment and considering ACR ‘exclusion’ and ‘association’ factors (i.e., their absence favors attribution to SLE), as well as ‘SLE-favoring factors’ of the Italian Study Group on NPSLE validated attribution model were also considered [66]. Clinical assessment and blood sampling were performed during routine clinic visits. The samples were collected after informed consent, following the ethics guidelines outlined in Section 2.1.

### 2.6. Peripheral Blood Mononuclear Cell (PBMC) Culture

PBMCs were purified from whole blood by Ficoll gradient (Cederlane, Hornby, ON, Canada), resuspended in 2 mL of RPMI-1640 (Euroclone, Pavia, Italy) supplemented with 2 mM L-Glutamine, 100 U/mL Penicillin G, 100 ug/mL Streptomycin and 20% FCS (Euroclone, Pavia, Italy) (RPMI-20%FCS) and counted.

Natural killer cells were separated from peripheral blood samples using the negative magnetic cell separation (MACS) system (Miltenyi Biotech, Gladbach, Germany) [67]. The analysis of purified cell fraction by flow cytometry with CD3-PerCp-Cy5.5, CD56-FITC moAbs (e-Bioscience, Frankfurt, Germany), demonstrated that the NK cell content was >90% (data not shown). NK cells were resuspended at 2 × 10^6^ cells/mL in 20 mL of RPMI 1640 (BioWhittaker, Verviers, Belgium) containing 10% human AB serum (Mediatech, Herndon, VA, USA), 1 mM non-essential amino acids, 2 mM glutamine, 1 mM pyruvate, 20 mM HEPES, 100 U/mL Penicillin and 100 μg/mL Streptomycin (Gibco BRL Life Technologies, Gaithersburg, MD, USA). Cell suspensions were stimulated with 100 U/mL of IL-2 (Hoffmann-LaRoche) on day 0 and cultured for 5 to 6 days at 37 °C, 5% CO_2_. NK cells were separated into KIR2DL2 expressing and non-expressing KIR2DL2 cells using CELLection™ Dynabeads (ThermoFisher; Milan, Italy) coated with anti-KIR2DL2 biotinylated antibody (BPS-Bioscience, San Diego, CA, USA). The efficiency of the separation was assessed using flow cytometry.

T cells were separated from peripheral blood samples using the positive magnetic cell separation (MACS) system Pan T Cell Isolation Kit (Miltenyi Biotech, Gladbach, Germany). The analysis of purified cell fraction by flow cytometry with CD2-PE and CD3-FITC (e-Bioscience, Frankfurt, Germany) demonstrated that the T cell content was >90% (data not shown). T cells were resuspended at 2 × 10^6^ cells/mL in 20 mL of Gibco CTS OpTmizer Pro SFM (ThermoFisher; Milan, Italy) and cultured for 5 to 6 days at 37 °C, 5% CO_2_. T cells were activated with plate bound 0.25 μg/mL antiCD3ϵ plus soluble antiCD28 in the presence or absence of 100 pM TGF-β1 (R&D Systems, Minneapolis, MN, USA) typically for 24 h.

### 2.7. T Cell Protein Transfection

T cells were transfected using the Pierce Protein Transfection Kit (ThermoFisher; Milan, Italy) following product instructions. A total of 4 × 10^5^ cells were transfected with 1 μg of peptide (U24, MBP). Transfection was performed for 3–4 h at 37 °C in 1 mL medium without fetal bovine serum (FBS). After transfection, a volume of complete medium with 20% FBS was added to each well. T cells treated with transfection reagent alone or transfected with 0.5 μg control fluorescent antibody (provided in the kit) were used as negative and efficiency control, respectively. A mean transfection efficiency of 95% for all the peptides was obtained (data not shown).

### 2.8. Lactate Dehydrogenase (LDH) Assay

LDH assay was performed to evaluate the effect of the transfection with U24 or MBP peptides in T cells. Transfected T cells were suspended at 5 × 10^4^ cells/mL and cultured for 4 h on a 96-well microplate at 37 °C with 5% CO_2_. A colorimetric-based lactate dehydrogenase (LDH) assay (Cytotoxicity Detection KitPLUS; Basel, Switzerland) was used, according to the manufacturer’s instructions. The percentage of viable cells was evaluated using the following equation: 100 − [(Culture Media LDH (OD) of treated cells − Culture Media LDH (OD) of control cells) × 100].

### 2.9. Proliferation Analysis

EdU assay (Abcam; Cambridge, UK) was performed to evaluate the proliferation of T cells. T cells were treated with fixative solution and incubated for 15 min, then with permeabilization buffer and incubated for 15–20 min. The reaction mixture was added to fluorescently label EdU and incubated for 30 min. The cells were analyzed with flow cytometer (FACS CantoII flow cytometer) and FlowJo software (Becton Dickinson, San Jose, CA, USA), acquiring 10,000 events.

### 2.10. Smad2 and TGF-β1 Analysis

Total and phosphorylated human Smad2 and TGF-β1 levels were evaluated with Human Smad2 ELISA Kit (Abcam; Cambridge, UK), Human Smad2 [pSpS465/467] (ThermoFisher; Milan, Italy) and Human TGF-beta 1 (R&D System; Minneapolis, MN, USA), following the manufacturer’s procedures.

### 2.11. Nedd4L

cDNA was reverse-transcribed from 8.0 µg total RNA using M-MLT RT (Invitrogen Corp., Carlsbad, CA, USA). Real-time monitoring of PCR was performed using the LightCycler System (Roche Applied Science, Indianapolis, IN, USA) and SYBR-Green I dye (Roche Diagnostics) as described previously [68]. Primer sequences and PCR conditions are listed in Table S2, Supplementary Information. The relative expression levels of these genes were obtained by normalizing mRNA expression to glyceraldehyde-3-phosphate dehydrogenase (GAPDH) mRNA expression as an endogenous control in each sample.

RNAi-mediated knockdown of Nedd4L was obtained with Assay ID 136860 (ThermoFisher; Milan, Italy) following manufacturer procedures.

### 2.12. Stable Cell Lines

To produce 721.221-ICP47-C1 and A2 cells, human HLA-C1 or HLA-A2 cDNAs were cloned into the pQCXIP retroviral vector (Clontech, Mountain View, CA, USA). This vector was cotransfected with pVSV-G (Clontech) into GP2-293 cells and supernatant was harvested two days post-transfection. The supernatant was centrifuged in Ultracel 50k filter centrifuge tubes (Millipore) to yield concentrated VSV-G pseudotyped MLV-based particles. 721.221-ICP47 cells, a derivative of the MHC class I-deficient 721.221 cell line [69] that expresses HSV-1 ICP47 to inhibit the transporter associated with antigen processing (TAP) complex, were transduced by incubation with concentrated virus for 3 h at 37 °C. Three days later, cells were placed under selection with 0.4 μg/mL puromycin (Invitrogen, Waltham, MA, USA).

### 2.13. Granzyme B ELISPOT Assay

Granzyme B secretion was measured using the GrB ELISPOT assay. Briefly, MultiScreen-IP plates (PVDF membrane, Millipore, Bedford, MA, USA) were coated overnight at 4 °C with 100 μL/well of anti-human GrB antibody (7.5 μg/mL in PBS, clone GB-10, PeliCluster, Cell Sciences, Norwood, MA, USA). Effector cells (100 μL/well) were added to triplicate wells at specified concentrations followed by 5 × 104 target cells per well (100 μL). After the specified effector-target cell incubation, the plates were washed and 100 μL/well of biotinylated anti-human GrB detecting antibody (0.25 μg/mL in PBS/1% BSA/0.05% Tween 20, clone GB-11, PeliCluster, Cell Sciences) was added. Plates were incubated for 3 h and 50 μL of Streptavidin-Alkaline Phosphatase (1:1500 in PBS/1% BSA, Gibco BRL Life Technologies, Gaithersburg, MD, USA) was added for 1 h. Spots were visualized with 100 μL/well of BCIP-NBT phosphatase substrate (KPL, Gaithersburg, MD, USA) and subjected to automated evaluation using the ImmunoSpot Imaging Analyzer system (Cellular Technology Ltd., Cleveland, OH, USA).

### 2.14. Calcein Acetoxymethyl Ester (CAM) Cytotoxicity Assay

721.221-ICP47-A2 and 721.221-ICP47-C1 cells were incubated overnight at 26 °C with peptides in Hybridoma-Serum Free Medium (Invitrogen) to stabilize cell surface A2/C1-peptide complexes. An aliquot of 2 × 10^5^ peptide-pulsed cells were then stained with a PE-conjugated pan-MHC class I specific antibody (clone W6/32; Dako, Agilent, Santa Clara, CA, USA) to verify the surface stabilization of MHC class I molecules. The remaining peptide-pulsed cells were stained with CAM (Invitrogen) at a 1:100 dilution for 1 h at 26 °C. CAM-stained cells were washed and then incubated with KIR2DL2 positive or negative NK cells for 4 h at different E:T ratios at 26 °C. The release of CAM into the supernatant was measured using a fluorescent plate reader (excitation 485 nm, absorption 530 nm). Percent specific lysis was calculated as (test release − spontaneous release)/(maximum release − spontaneous release).

### 2.15. Cytometric Analysis and CD107a Degranulation Assay

NK cells obtained in the different culture conditions from 20 controls and 12 MS patients were characterized with a specific anti-CD panel (CD3-PerCp-Cy5.5, CD56-FITC, CD107a-PE) (e-Bioscience, Frankfurt, Germany), anti-KIR2DL2-2DS2-2DL3/CD158b-PE (ThermoScientific, Erembodegem, Belgium) monoclonal antibodies. Samples, incubated with the moAbs for 30 min in ice and washed, were analysed with FACS CantoII flow cytometer and FlowJo software (Becton Dickinson, San Jose, CA, USA), acquiring 10,000 events. Lymphocytes were identified according to forward/side scatter profile and NK cells (CD3-/CD56+) were defined and gated within the lymphocyte gate. CD158b levels were measured in the CD3-/CD56+ gated cells. Cell viability was assessed by propidium iodide staining. Anti-isotype controls (Exbio, Praha, Czech Republic) were performed. For the CD107a degranulation assay, after 1 h of incubation at 37 °C and 3 h of treatment with Golgi Stop solution (Becton Dickinson, San Jose, CA, USA), PBMCs were stained with anti-CD3 and anti-CD56 moAbs. Ten thousand events were acquired.

### 2.16. Isothermal Titration Calorimetry Analysis

After dialysis (48 h), the cleaved hNedd4L-WW3* domain in 10 mM sodium phosphate buffer, pH 7.45, was used for ITC experiments. The dialysis buffer alone was utilized to measure heats of dilution and to dissolve the U24 peptides. The sample was then concentrated to 40 to 150 μM, depending on the estimated *K_d_* value, using Microsep centrifugal devices with a MWCO of 1 kDa. The concentration of the sample was determined by the absorbance at 280 nm in the dialysis buffer on a Nanodrop UV-Vis spectrophotometer, and calculated using the theoretical extinction coefficient obtained from the ProtParam tool. A 15× to 30× concentrated peptide stock solution was made by dissolving the purified peptides, U24-6A, U24-6B and pU24-6B, in the same buffer as the protein and its pH was adjusted to match the buffer and hNedd4L-WW3* protein within ±0.02. The peptide amounts were determined gravimetrically and concentrations adjusted according to the absorbance at 280 nm in the dialysis buffer. Both protein and peptide stock solutions were filtered and degassed for 10 min before loading into the sample cell and injection syringe, respectively.

ITC experiments were performed on a MicroCal iTC200 (GE Healthcare) at 25 °C. The titration protocol consisted of a preliminary injection of 0.2 μL of the peptide solution, followed by 19 consecutive 2 μL injections into the sample cell (200 μL) containing the hNedd4L- WW3* domain studied. The time between each injection was 300 s for U24-6A and U24-6B peptides and 180 s for pU24-6B peptide. Control titrations of the peptide solution into protein-free dialysis buffer were completed. The heats of dilution for the peptide were subtracted from the original heats prior to data fitting to a one-to-one bimolecular interaction model to obtain *K_a_* and Δ*H*°, at a binding stoichiometry *n* of 1.0 (adjustable parameter). The ITC experiments were independently repeated three times, with mean values and standard deviations reported.

### 2.17. ^1^H−^15^N HSQC NMR Titrations and K_d_ Calculations for the Fyn-SH3 Protein and U24 Peptide Interaction

Solutions of ^15^N-Fyn-SH3 were prepared by three successive rounds of concentration and dilution with NMR buffer [10 mM sodium phosphate, 10% D_2_O, 0.5 mM benzamidine, and 0.1% sodium azide (pH 6.0)] using a 15 mL regenerated cellulose centrifugal filter device with a MWCO of 3000 Da (Millipore). The final protein concentration was determined using an extinction coefficient (ε_280_) of 16,960 cm^−1^M^−1^ (Protparam; http://www.expasy.ch, accessed 15 August 2020). The protein solutions (final concentrations = 0.2–0.6 mM) were then transferred to a 5 mm NMR tube, and a ^1^H−^15^N HSQC spectrum was recorded at 25 °C using a Bruker Avance III 600 MHz NMR spectrometer (Milton, ON, Canada) equipped with a TCI cryoprobe. Small volumes of unlabeled U24-6B and pU24-6B peptide stock solutions, with concentrations of 12 mM and 16 mM, respectively. HSQC spectra were recorded after each subsequent addition. A total of 14–15 different protein:peptide ratios between 1:0 and 1:16 for pU24-6B peptide and 1:31 for U24-6B were examined. The pH of the solution was measured to be 6.0 at the start of the titration and found to be identical after the last addition of peptide. The amide chemical shifts of Fyn-SH3 were assigned based on previously published assignments by Mal et al. [70], as described by Sang et al. [29]. The dissociation constant (*K_d_*) was calculated according to the method described by Williamson et al. [71].

### 2.18. ELISA Assay for U24, pU24 and MBP IgG Titration

U24, pU24 and MBP specific IgG titration was performed on plasma samples using 96-wells plates coated with 5 μg/mL of U24, pU24 or MBP peptide in bicarbonate buffer pH 9.6. Briefly, after peptides coating overnight at 4 °C, the plate was washed three times with PBS-Tween-20 0.05% and saturated with PBS with 3% BSA for 1 h and 30 min at 37 °C. Plasma samples were serially diluted (1:20, 1:40, 1:60, 1:100, 1:160, 1:240, 1:320) in PBS with 3% BSA and the 50 μL were added to each well for 1 h and 30 min at 37 °C. The detection was performed by adding 100 μL of anti IgG-HRP (Bethyl, Montgomery, TX, USA) diluted 1:10,000 for 1 h and 30 min at 37 °C, followed by 50 μL of TMB. The plate was read at 450 nm. Titer was defined as the last dilution showing an optical density greater than average negative control plus 3 standard deviations.

### 2.19. Statistics

Statistical analysis was performed with Stat View (SAS Institute Inc., Cary, NC, USA). Biological data presented a parametric distribution (Kolmogorov-Smirnoff test) and were analyzed by One way ANOVA for multiple comparison and the *p* values corrected for multiple comparisons, by Bonferroni’s correction. Percentages were compared by Fisher exact test. Statistical significance was assumed for *p* < 0.05 (two tailed).

## 3. Results

### 3.1. Endocytic Recycling Model: Interactions with hNedd4L-WW3*

#### 3.1.1. U24-6A and -6B Bind hNedd4L-WW3* with Micromolar Affinity

**Figure 1:**
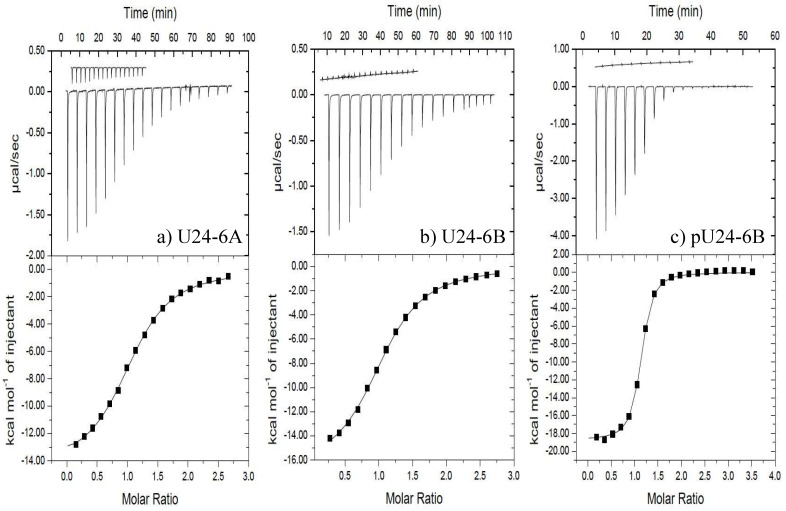
ITC data for (**a**) U24-6A peptide, (**b**) U24-6B peptide, and (**c**) pU24-6B peptide binding to hNedd4L-WW3* at 25 °C. In each case, at least two additional runs were performed and looked nearly identical to the ones shown here. (**a**) (Upper) Raw titration data for nineteen 2 μL injections of 0.95 mM U24-6A peptide into the ITC cell containing 0.07 mM hNedd4L-WW3* in 10 mM sodium phosphate pH 7.45. (**b**) (Upper) Raw titration data for nineteen 2 μL injections of 0.51 mM U24-6B peptide into the ITC cell containing 0.034 mM hNedd4L-WW3* in 10 mM sodium phosphate pH 7.45. (**c**) (Upper) Raw titration data for nineteen 2 μL injections of 1.15 mM pU24-6B peptide into the ITC cell containing 0.068 mM hNedd4L-WW3* in 10 mM sodium phosphate pH 7.45. (**a**–**c**) (Upper) Insets show heat of dilution data. (Lower) Integrated heat data (points) and best fit (line) to a “one set of sites” model (*n* = 1; adjustable parameter).

In order to investigate whether the increased positive charge in U24-6B (Table 1) changes the interaction with hNedd4L-WW3*, ITC experiments were conducted using peptides representing the first fifteen residues of U24-6B and U24-6A. The peptides were synthesized and hNedd4L-WW3* was expressed and purified in house. Resulting representative ITC binding isotherms are shown in Figure 1a,b, along with heat of dilution data. In total, three independently performed ITC experiments were conducted for each peptide. The resulting average thermodynamic parameters obtained are summarized in Table 2. Overall, the data showed that the binding affinity for both U24-6A and -6B is nearly identical. Previously, Sang et al. [30] had found *K_d_* to be 6.3 ± 0.3 μM, which is not statistically significantly different to that found here. Here, a slightly lower value for Δ*H*° for the U24-6B/hNedd4L-WW3* interaction was recorded, suggesting that the positive charge arising from Arg3 (Table 1) contributes to a more exothermic interaction, most likely through increased electrostatic interactions. The decrease in the *ΔS*° value, on the other hand, indicates a more ordered state, possibly reflecting an ordering of solvating water around the charged Arg side-chain. Overall, however, the Δ*G*° is the same for both U24-6A and -6B, indicating an enthalpy-entropy compensation.

#### 3.1.2. Phosphorylation at Thr6 of U24-6B Enhances the Affinity with hNedd4L-WW3*

Like most viral proteins, U24 is extensively post-translationally modified (PTM) when expressed in eukaryotes. The size of U24 expressed in T-cells and observed on SDS-PAGE gels [41] was found to be two times larger than the expected molecular weight of 10 kDa (based on amino acid sequence). The exact modifications were, however, not identified, but phosphorylation is certainly a possible PTM. Indeed, it has been shown that recombinantly expressed U24 from HHV-6A can be phosphorylated at Thr6 [28]. In addition, previous work by Sang et al. [30] had shown that phosphorylated U24-6A binds very strongly (*K_d_* = 0.76 ± 0.03 μM; Δ*H*° = -58.7 ± 0.4 kJ.mol^−1^; Δ*S° =* -79.8 ± 0.9 J. mol^−1^.K^−1^; Δ*G*° = (−3.49 ± 0.05)×10^1^ kJ.mol^−1^) to hNedd4L-WW3* domain. This fact, as well as the finding that U24-7 also displayed a high binding affinity to WW3*, with a *K_d_* = 1.22 ± 0.01 μM, suggested that the presence of a positive and negative charge in close proximity helped to favour binding, i.e., the Arg5/pThr6 in pU24-6A might present similar features as the His3/Glu4 pair found in U24-7. In order to examine the effect of adding a positive charge at position Arg3, ITC experiments were conducted to quantify the interaction between pU24-6B and hNedd4L-WW3. A peptide representing the first fifteen residues (Table 1) was used in the study and purchased as a trifluoroacetic acid free compound. The WW3* domain was prepared as previously described.

A resulting representative ITC binding isotherm is shown in Figure 1c. As before, three independently performed experiments were conducted. The resulting average thermodynamic parameters obtained are summarized in Table 2 and showed an order of magnitude lower *K_d_* for pU24-6B, as compared to U24-6B. Similarly to what was observed for the unphosphorylated peptides, the data showed that the binding affinity for both pU24-6A and -6B is not statistically significantly different. The slightly lower values found for Δ*H*° for the pU24-6B/hNedd4L-WW3* interaction again suggests that the positive charge arising from Arg3 results in more exothermic interactions, most likely due to the increased electrostatic interactions. Additionally, the decrease in the Δ*S*° value again indicates a more ordered state. The Δ*G*° is the same for both pU24-6A and p-U24-6B, indicating that enthalpic and entropic effects compensate each other.

#### 3.1.3. Phosphorylated U24-6A and -6B Affect T-Cell Proliferation via Nedd4L Knockdown

**Figure 2:**
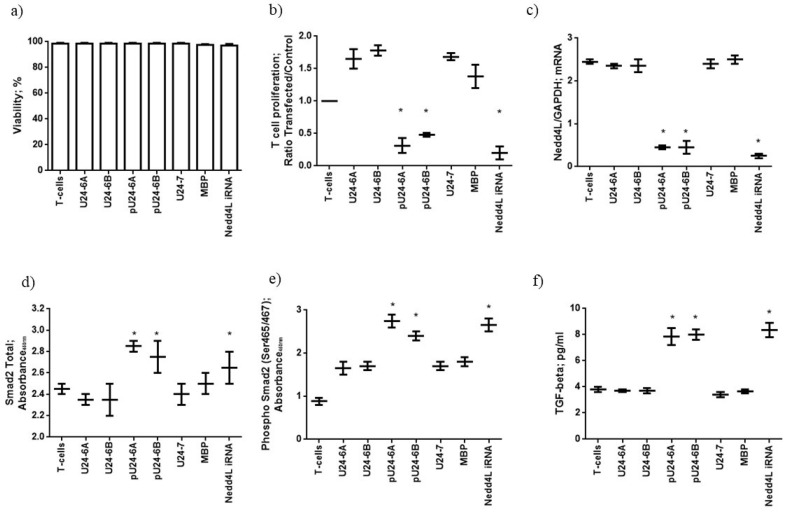
(**a**) T cells from 12 MS patients were purified and transfected with the transfection reagent alone (T cells) the peptides U24-6A, U24-6B, pU24-6A, pU24-6B, MBP or U24-7 or Nedd4L iRNA. T-cell viability was evaluated using a lactate dehydrogenase (LDH) assay. (**b**) T-cell proliferation was evaluated using an EdU assay. (**c**) Levels of Nedd4L mRNA in comparison with GAPDH mRNA levels. Levels of (**d**) total Smad2 and (**e**) phosphorylated Smad2 and (**f**) TGF-β1, performed by ELISA assay. The values are reported as mean ± SD. * significant *p* value < 0.05, one way ANOVA for multiple comparison corrected for multiple comparisons, by Bonferroni’s correction. iRNA: RNA-mediated knockdown of Nedd4L.

To assess the possible effect of U24 on Nedd4L expression in MS, we transfected T cells from 12 MS patients with U24 or MBP peptides, to mimic the expression these proteins inside HHV-6 infected T cells. T-cell viability, evaluated using a lactate dehydrogenase (LDH) assay, was not affected by peptide transfection (Figure 2a). We assessed T-cell proliferation status after protein transfection. The transfection with U24-6A, U24-6B, U24-7 and MBP induced T-cell proliferation, while pU24-6A (p: 0.003; p_c_: 0.021; One way ANOVA) and pU24-6B (p: 0.007; p_c_: 0.049) significantly decreased T-cell proliferation (Figure 2b). To pinpoint the possible implication of Nedd4L in the inhibition of T-cell proliferation via U24, the levels of Nedd4L mRNA expression were assessed in T cells. The transfection with U24-6A, U24-6B, U24-7 and MBP did not modify Nedd4L mRNA expression, while pU24-6A (p: 0.0025; p_c_: 0.018) and pU24-6B (p: 0.004; p_c_: 0.028) significantly decreased Nedd4L mRNA expression (Figure 2c), comparable to the effect of a Nedd4L RNAi-mediated knockdown (p: 0.0015; p_c_: 0.011) (Figure 2c). Since the effect of the Nedd4L RNAi-mediated knockdown on T-cell proliferation is like the inhibition observed after pU24-6A and pU24-6B transfection, this might suggest that pU24-6A/pU24-6B reduce Nedd4L expression with a consequent reduction of T-cell proliferation. Nedd4L controls the activity of TGF-β1 (Tumour growth factor-beta1), which in turn supports T-cell development, homeostasis, tolerance, and differentiation [72]. Nedd4L inhibits TGF-β1 signaling by triggering Smad2 and TGFBR1 ubiquitination and proteasome-dependent degradation. As a proof of concept, we evaluated the levels of total and phosphorylated Smad2 protein and TGF-β1, under the same culture conditions. An increased expression of total and phosphorylated Smad2 protein and TGF-β1 secretion were observed in Nedd4L RNAi ((total Smad2 = p: 0.004; pc: 0.028), (phosphorylated Smad2 = p: 0.0026; p_c_: 0.018), (TGF-β1 = p: 0.0019; p_c_: 0.013)), pU24-6A ((total Smad2 = p: 0.0027; pc: 0.019), (phosphorylated Smad2 = p: 0.0015; p_c_: 0.011), (TGF-β1 = p: 0.0023; p_c_: 0.016)), and pU24-6B ((total Smad2 = p: 0.0034; pc: 0.024), (phosphorylated Smad2 = p: 0.0039; p_c_: 0.027), (TGF-β1 = p: 0.0026; p_c_: 0.018)) conditions (Figure 2d–f). These data suggest that pU24-6A and pU24-6B act like Nedd4L RNAi in reducing Nedd4L mRNA expression and enhancing TGF-β1 secretion via Smad2 phosphorylation.

### 3.2. Mimicry Model: Interactions with Fyn-SH3

#### 3.2.1. U24-6B Has a Higher Binding Affinity for Fyn-SH3 Than U24-6A

To further understand the role of U24 in MS, binding interactions with Fyn-SH3 were investigated in order to determine the validity of the MBP mimicry hypothesis. As detailed in the introduction, MBP is an important component of the myelin sheath and it has many additional functions such as cytoskeletal turnover at leading edges of membrane ruffles [73], as well as being involved in Fyn-SH3-mediated signaling pathways during myelin formation. The specific interaction between MBP and Fyn-SH3 has been found to be important in myelination and to be dependent on the phosphorylation state of MBP [33,74].

Sang et al. previously investigated the interaction between a peptide representing the first fifteen residues of U24-6A with Fyn-SH3, using both NMR and ITC [29]. The dissociation equilibrium constant was found to be *K_d_* = 5.1 ± 0.3 mM, i.e., the interaction between both partners is quite weak. Interestingly, removal of the first two residues in the U24-6A sequence, i.e., Met1 and Asp2 (Table 1), resulted in a *K_d_* of 1.9 ± 0.1 mM [75] (Figure S2). To date, no studies have examined binding between U24-6B and Fyn-SH3, nor what effect phosphorylation at Thr6, which is located in the PxxP motif, might have on this interaction.

**Figure 3:**
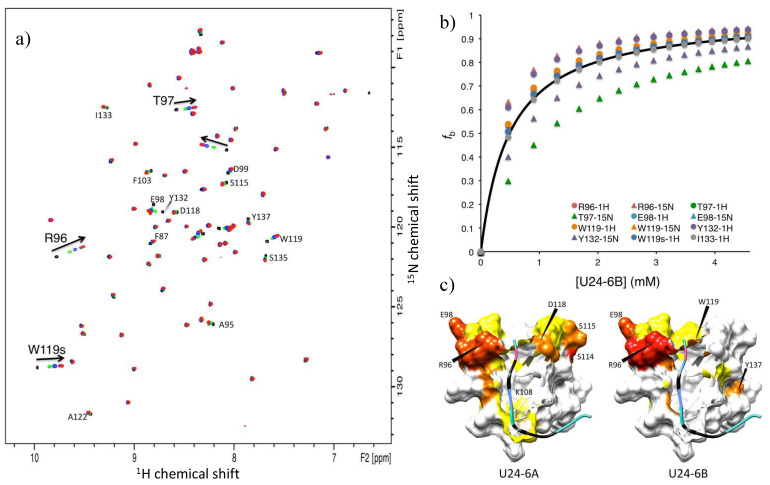
(**a**) Overlay of 2D ^1^H-^15^N HSQC spectra of 0.2 mM ^15^N-labelled Fyn-SH3 in 10 mM sodium phosphate, 10% D_2_O, 0.5 mM benzamidine, and 0.1% sodium azide (pH 6.0), recorded at 25 °C using a Bruker Avance III 600 MHz NMR, as a function of increasing U24-6B peptide concentration: (black) 1:0, (green) 1:2, (blue) 1:4, (purple) 1:8 and (red) 1:13 Fyn-SH3/U24-6B molar ratio. Most perturbed resonances (with the exception of four) could be assigned, as designated by the labels indicating residue type and number found next to the peaks (“s” indicates side-chain). (**b**) Fraction bound as a function of U24-6B peptide concentration (symbols, as indicated in the legend). The curve represents the calculated values of fraction bound for a K_d_ = 0.5 ± 0.2 mM. (**c**) Chemical shift perturbations mapped onto model 4 of 1A0N.pdb, i.e., Fyn-SH3 bound to PI3-kinase peptide 2. For U24 ligands, Pro is in black, Arg in light blue, Asp in pink and the PxxP motif is represented as an edged ribbon. The color codes for the chemical shift perturbations are: $\Delta \delta_{norm}=0.80-1.00$ in red, $\Delta \delta_{norm}=0.60-0.79$ in orange red, $\Delta \delta_{norm}=0.40-0.59$ in orange, $\Delta \delta_{norm}=0.20-0.39$ in yellow and values < 0.20 in white, where $\Delta \delta_{norm}=\Delta \delta /\Delta \delta_{max}$, with $\Delta \delta =\sqrt{{\left(\delta_{bound,H}-\delta_{apo,H}\right)}^{2}+0.1{\left(\delta_{bound,N}-\delta_{apo,N}\right)}^{2}}$ and $\Delta \delta_{max}$ being the largest value found for a given residue i.

NMR titration experiments of U24-6B peptide added to ^15^N-labelled Fyn-SH3 were conducted. As the overlay of ^1^H-^15^N NMR HSQC spectra shows (Figure 3a), a number of residues were perturbed. The chemical shift changes of these residues were tabulated and the fraction bound was calculated, according the method used by Zarrine-Afsar et al. [76] (Figure 3b), using the *K_d_* values obtained according to the method described by Williamson et al. [71]. Interestingly, the average *K_d_* obtained from 12 shifts was 0.5 ± 0.2 mM. In addition, chemical shift perturbations were mapped onto the Fyn-SH3 structure deposited in the Protein Databank under 1A0N.pdb (Figure 3c) and indicated that while both U24-6A and U24-6B consistently perturbed residues in the RT loop of Fyn-SH3, i.e., R96 and T97, the peripheral residues affected were different for the two ligands. Noticeably, the large region located around residues 114-118 in Fyn-SH3 is highly perturbed by U24-6A, but not at all by U24-6B. In contrast, Y137 is affected by U24-6B but not U24-6A. Overall, the data obtained from NMR suggests that the addition of the positive side-chain at position 3 in the U24 sequence strengthens the binding affinity between U24-6B and Fyn-SH3. The chemical shift perturbations observed also suggest that the mode of binding may be different between U24-6A and U24-6B.

#### 3.2.2. Phosphorylation of U24 Lowers Binding Affinity to Fyn-SH3

**Figure 4:**
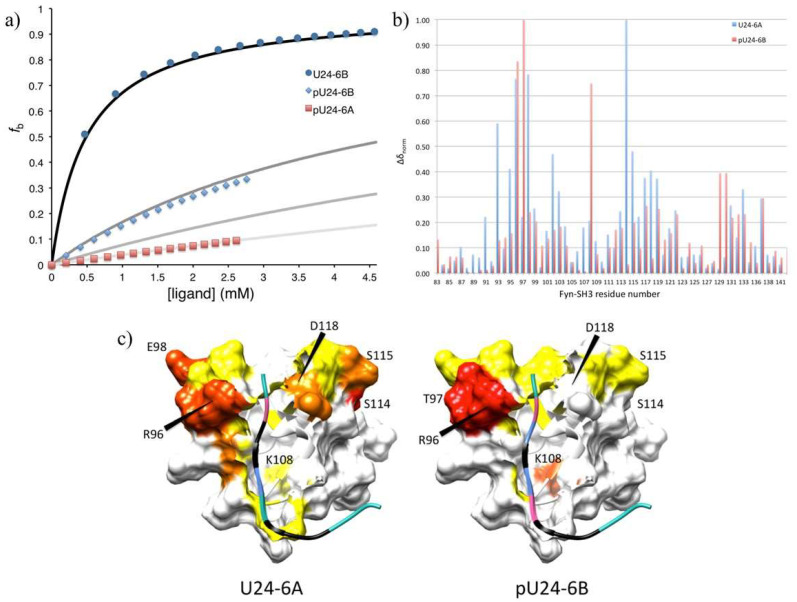
(**a**) Comparison of the fraction bound as a function of ligand concentration for peptide ligands U24-6B, pU24-6B and pU24-6A (as indicated in the legend), based on the ^1^H chemical shift of W119s. The curves in black to light grey represent calculated *f*_b_ values for K_d_’s of 0.5 mM (black), 5 mM (dark grey), 12 mM (grey), and 25 mM (light grey). In the case of pU24-6A where binding is weakest, the data fit equally well to a K_d_ of 12 mM (red squares) and 25 mM (fit to data not shown here), which is why both grey and light grey lines are illustrated here. (**b**) Normalized chemical shift perturbations for residues in Fyn-SH3 for U24-6A (blue bars) and pU24-6B (red bars). The values for $\Delta \delta_{norm}$ are calculated as described in Figure 3. Both ligands interact with R96 and T97 in the RT loop of Fyn-SH3. Interactions with subsequent residues are different between U24-6A and pU24-6B. (**c**) Chemical shift perturbations mapped onto model 4 of 1A0N.pdb. For the U24 ligands, the prolines are shown in black, arginines in light blue, negatively charged amino acids (i.e., Asp and phospho-Thr6) in pink and the PxxP motif is represented as an edged ribbon. The color codes for the chemical shift perturbations are described in Figure 3. Figure generated using CHIMERA [77].

What effect phosphorylation at Thr6 might have on binding affinity was examined using NMR titrations with peptides representing the first fifteen amino acids of U24 with a phospho-threonine at position 6, i.e., pU24-6A and pU24-6B (Table 1), and ^15^N-labelled Fyn-SH3. Saturation was not achieved for both pU24-6A and -6B, in part due to the limited amount of sample available, but also because the *K_d_* for the interaction with Fyn-SH3 is much larger. As seen in Figure 4a, the plateau values were not observed for pU24-6B and pU24-6A, making a precise determination of *K_d_* difficult. Nevertheless, values for the binding affinity could be estimated, based on a number of resonance shifts: *K_d_* ≥ 5 mM for pU24-6B, whereas *K_d_* ≥ 12 mM for pU24-6A. Figure 4a illustrates the data for the ^1^H resonance of the Trp side-chain, W119s. For both phosphorylated peptides, the trends clearly indicate that, as with MBP, phosphorylation weakens the interaction between U24 and Fyn-SH3.

In order to further characterize where phosphorylated peptides bind to Fyn-SH3, the chemical shift changes, $\Delta \delta_{norm}$, as described in the caption of Figure 3, were plotted as a function of residue number for U24-6A (based on values reported in Sang’s thesis [75]) and pU24-6B. As Figure 4b clearly shows, the amino acids that are most perturbed in Fyn-SH3 are different, depending on whether the ligand is U24-6A or pU24-6B. While in both cases shifts in the RT loop binding site are observed, perturbations around residue 114 upon titrations with U24-6A were shifted to residue 108 when the ligand is pU24-6B.

Mapping the observed chemical shift changes onto model 4 of 1A0N.pdb helped clarify why different residues are perturbed upon binding of U24-6A versus pU24-6B. As illustrated in Figure 4c, the perturbation of K108 in Fyn-SH3 upon binding of pU24-6B is consistent with the close proximity of the phosphorylated Thr6, shown in pink in the model on the right. On the other hand, the lack of perturbations for residues 114-118 in Fyn-SH3 upon binding of pU24-6B as compared to U24-6A is not easily accounted for with this model.

Overall, the data presented in this section shows that phosphorylation does not lead to a stronger interaction between proline-rich segments and Fyn-SH3. Although the contact to K108 is strengthened for pU24-6B (i.e., orange versus yellow, Figure 4c), there are very few residues around this amino acid that interact with the ligand, thereby possibly accounting for the weakened interaction with Fyn-SH3 upon phosphorylation of Thr. The model presented in Figure 4c (right) could serve as a first structural model to explain why phosphorylation of MBP leads to a weakened interaction with Fyn-SH3 at a molecular level.

### 3.3. Immune Control Model: Interaction with KIR Receptors

#### 3.3.1. pU24-6A Controls NK Cell Activation

**Figure 5:**
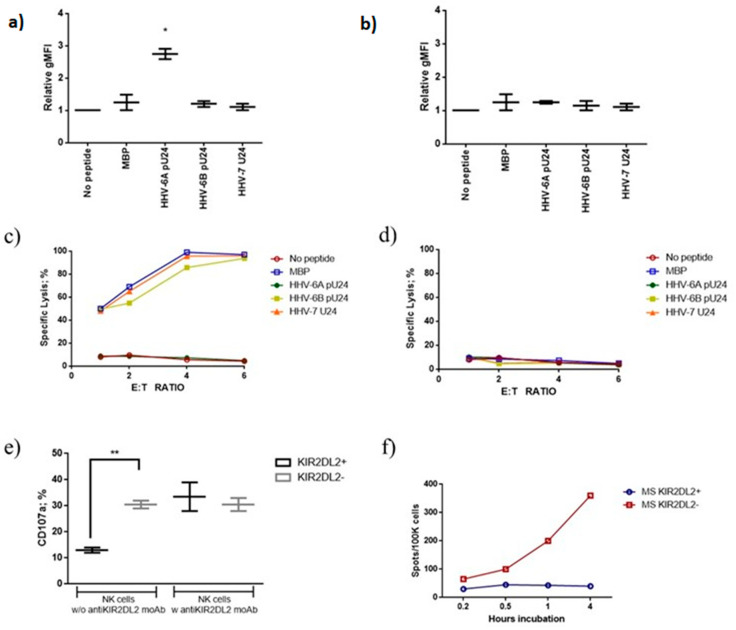
Expression of (**a**) HLA-C1 in 721.221-ICP47-C1 and (**b**) HLA-A2 in 721.221-ICP47-A2 after MBP, pU24-6A, pU24-6B or U24-7 treatment. gMFI: The data are presented as mean ± SD. * significant *p* value, Student *t* test. (**c**) 721.221-ICP47-C1 or (**d**) 721.221-ICP47-A2 cells were incubated with the different peptides and co-cultured with KIR2DL2 positive NK cells from MS patients. The cytolytic activity of KIR2DL2 positive NK cells was tested by calcein acetoxymethyl ester (CAM) cytotoxicity assay. The data are presented as mean. (**e**) NK cells from MS patients were co-cultured with 721.221-ICP47-C1 cells incubated with pU24-6A. The expression of CD107a, marker of NK cell activation, was evaluated in the absence (w/o) or presence (w) anti-KIR2DL2 antibody. The data are presented as mean ± SD. * significant *p* value, one way ANOVA for multiple comparison corrected for multiple comparisons, by Bonferroni’s correction. ** significant *p* value, Fisher exact test. (**f**) Granzime B Fluorospot assay on KIR2DL2 positive and negative NK cells from MS patients co-cultured with 721.221-ICP47-C1 cells incubated with pU24-6A as a function of an increasing incubation time (0.2, 0.5, 1, or 4 h at 37 °C).

Previous work had shown that NK cells from MS patients have an increased susceptibility to HHV-6 infection, in particular in the presence of KIR2DL2 receptor [78]. Phosphorylated U24 has an amino acid sequence motif with an elevated half time of dissociation (HTD: 24) from HLA-C1 molecules (estimated by www.bimas.cit.nih.gov/cgi-bin/molbio/ken_parker_comboform, accessed on 20 September 2022), which are ligands for KIR2DL2 [47]. These data suggest a possible interaction of pU24 peptide with NK cells via KIR2DL2/pU24/HLA-C1. To evaluate the possible interaction of U24 with NK cells, we tested for the ability of the peptides to modify NK cell cytotoxicity and degranulation. We enrolled 12 MS Relapsing Remitting patients and primary NK cells from these patients were purified, expanded by IL-2 and IL-15 stimulation [47] and sorted into KIR2DL2 positive and KIR2DL2 negative NK cell subsets using anti-KIR2DL2 coated beads. These NK cells were then tested in cytotoxicity assays for recognition of an HLA-C1 or HLA-A2 expressing transporter associated with antigen processing (TAP)-deficient 721.221 cell line (721.221-ICP47-C1; 721.221-ICP47-A2) incubated with the peptides of interest. Because TAP is necessary for the translocation of peptides from the cytoplasm into the endoplasmic reticulum, MHC class I molecules expressed in these cells cannot load peptides derived from endogenously synthesized proteins, and are rapidly internalized from the plasma membrane [79]. Thus, most of the HLA-C1 and HLA-A2 molecules expressed by these cells are empty and can be loaded with exogenous peptides. Moreover, peptide binding to HLA-C1 or HLA-A2 can be assessed by staining for an increase in steady-state levels of MHC class I expression on the cell surface. An increased expression of HLA-C1 in the presence of pU24-6A (Figure 5a) (p: 0.0012; pc: 0.004; One way ANOVA) was observed, while pU24-6B, MBP and U24-7 presented a lower binding affinity for HLA-C1, resulting in a lower stability of HLA-C1 expression at the cell surface, suggesting a lower ability to stabilize HLA-C1 expression (Figure 5a). HLA-A2 expression was not modified by all the peptides tested (Figure 5b).

Given these results, the ability of pU24-6A, pU24-6B, MBP and U24-7 to modify the cytolytic activity of KIR2DL2 positive NK cells was tested. 721.221-ICP47-C1 or 721.221-ICP47-A2 cells were incubated with the different peptides and co-cultured with NK cells from MS patients. MBP, pU24-6B and U24-7 pre-incubation induced 721.221-ICP47-C1 cell killing (Figure 5c) (*p* < 0.001; Fisher exact test), while pU24-6A pre-incubation maintained KIR2DL2 positive NK cells unresponsive towards 721.221-ICP47-C1 cells ((Figure 5c) (p: NS; Fisher exact test). In contrast, no killing was observed with 721.221-ICP47-A2 pre-incubation with the tested peptides and the co-culture with KIR2DL2 positive NK cells (Figure 5d).

To further elucidate the possible role of the KIR2DL2/pU24/HLA-C1 interaction in controlling NK cell activation, KIR2DL2 positive and negative NK cells from MS patients were pre-treated with anti-KIR2DL2 antibody and then co-cultured with 721.221-ICP47-C1 cells incubated with pU24-6A. The expression of CD107a, marker of NK cell activation, was lower in KIR2DL2 positive NK cells in comparison with KIR2DL2 negative NK cells (*p* < 0.01; Fisher exact test) and was reported to levels comparable to KIR2DL2 negative NK cells by anti-KIR2DL2 antibody treatment (p: NS; Fisher exact test) (Figure 5e).

To evaluate the role of pU24-6A in modifying NK cell secretion of Granzime B (GrB), 721.221-ICP47-C1 cells were incubated with pU24-6A and co-cultured with KIR2DL2 positive NK cells from MS patients. GrB Fluorospot assays were then performed as a function of increasing incubation time (0.2, 0.5, 1, or 4 h at 37 °C). As the time of incubation increased, the resulting number of GrB spots increased linearly in MS patients negative for KIR2DL2 receptor (Figure 5f). On the contrary, MS patients positive for KIR2DL2 receptor presented a lower degranulation cell frequency, which was independent of incubation time (Figure 5f).

Overall, the data presented in this section showed for the first time a clear involvement of pU24-6A in NK cell activation.

#### 3.3.2. MS Patients Showed Increased Levels of U24 Derived Peptide IgG

**Figure 6:**
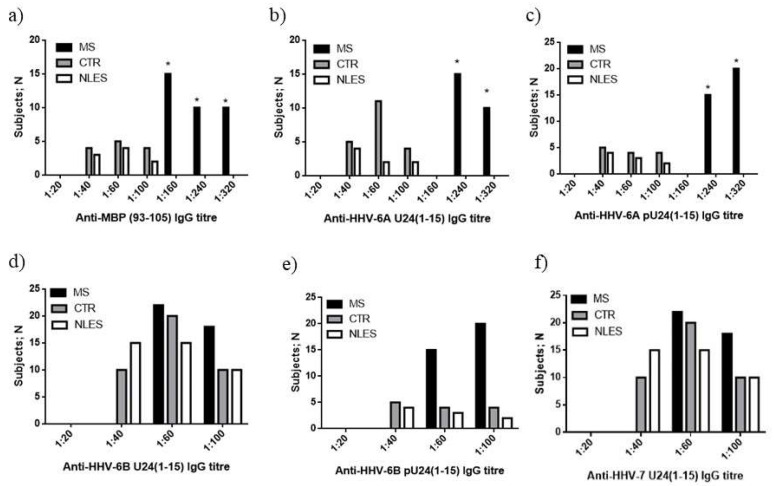
IgG titers, measured in the plasma samples of 40 MS—RRMS patients; 40 CTR—healthy subjects; 40 NLES—neurolupus patients, specific for (**a**) MBP_93-105_, (**b**) U24-6A_1-15_, (**c**) pU24-6A_1-15_, (**d**) U24-6B_1-15_, (**e**) pU24-6B_1-15_, and (**f**) U24-7_1-15_ (cf. Table 1 for sequences). * significant *p* value, Fisher exact test.

Based on the findings described above, it could be hypothesized that the presence of high levels of pU24-6A and pU24-6B in MS patients might affect T and NK cell immune response. As a proof of concept of the presence of higher levels of pU24-6A and pU24-6B in MS patients, we evaluated the titration of plasma IgG towards MBP and U24 peptides listed in Table 1, in 40 MS patients, 40 control subjects (CTR) and 40 neurolupus (NLES) patients. The plasma samples were serially diluted (1:20, 1:40, 1:60, 1:100, 1:160, 1:240, 1:320) and titers were defined as the last dilution showing an optical density greater than average negative control plus 3 standard deviations. MS patients showed the highest titers of IgG towards MBP and U24-6A in comparison with healthy patients and neurolupus patients (Figure 6a,b, respectively) (MBP p:0.001; U24-6A p: 0021). Interestingly, high titers of IgG specific for the phosphorylated form of HHV-6A U24 (pU24-6A) peptide were also observed in MS patients in comparison with controls (Figure 6c) (pU24-6A p: 0.0019). Moreover, we observed a trend to have a higher number of MS patients with high titers of IgG towards U24-6B, pU24-6B and U24-7 in comparison with controls and neurolupus patients (Figure 6d–f). These data suggest a higher immune response towards U24 and its phosphorylated form in MS patients, supporting a biological role of U24 during MS.

## 4. Discussion

Multiple sclerosis is a complex multifactorial disease. A number of key cells and proteins have been implicated as potentially playing a role [11,13]: from T cells, with their TCR/CD3 receptors on the cell surface, to KIR2DL2 and U24 from HHV-6A. In this contribution, we attempted to shed further light into the function of U24 by examining its interaction with a Nedd4L WW domain, Fyn-SH3 and KIR2DL2. The study focused in large part on U24 from HHV-6B, which shares an almost identical proline rich segment to U24-6A, save for one residue at position 3 (Table 1). Because many studies in the literature suggest that HHV-6A is more frequently associated with MS [8,36,38,41,54,80,81,82,83,84,85,86,87,88,89,90,91,92,93,94] than HHV-6B [27], directly comparing the interactions of U24-6B to U24-6A is informative.

The ITC results presented in Figure 1 showed surprisingly little difference in the interaction between U24-6A and hNedd4L-WW3* domain and its U24-6B counterpart. This was particularly striking in the case of the phosphorylated peptides, as it might have been anticipated that the introduction of an additional positive charge at residue 3 would perturb the balance of the positive/negative charge pair found to be important in the interaction of pU24-6A and U24-7 with hNedd4L-WW3*. However, upon examination of the predominant structural model found from molecular dynamics simulations reported by Sang et al. [30], the data observed here can be rationalized. As seen in Figure S3a, binding between U24-6A and hNedd4L-WW3* occurs predominantly between the PY motif and subsequent residues. The PxxP motif at the N-terminal end, which is preceded by Pro3 (indicated by the arrow) in U24-6A, points away from the binding groove in hNedd4L-WW3*. Given that the proline rich segment is known to adopt a PPII helix and that this structure is extended [95], changing Pro3 to Arg3 would not result in stronger interactions, as this part is positioned too far to reach the hNedd4L-WW3* domain. For the phosphorylated peptides, the structural model shown in Figure S3b again shows how a change from Pro3 to Arg3 would have little impact on the binding affinity. Interestingly, the phosphorylation of Thr6 adds a negative charge that comes into close proximity with R492 in hNedd4L-WW3*, thereby stabilizing the interaction and explaining the higher binding affinities observed. Indeed, R492 was found to be perturbed in NMR titrations for pU24-6A and hNedd4L-WW3* [75]. Overall, the reported findings suggest that there is little difference in the interaction between U24-6A or U24-6B and hNedd4L-WW3* domain, both in the unphosphorylated and phosphorylated forms. This would thus suggest that the PY motif of U24 functions similarly in both HHV-6A and -6B.

To assess the possible effect of U24 on Nedd4L expression in MS, we transfected T cells from MS patients with U24 or MBP peptides, to mimic the expression these proteins inside HHV-6 infected T cells. The transfection with U24-6A, U24-6B, U24-7 and MBP did not modify Nedd4L mRNA expression, while pU24-6A and pU24-6B significantly decreased Nedd4L mRNA expression, comparable to the effect of a Nedd4L RNAi-mediated knockdown. Since the effect of the Nedd4L RNAi-mediated knockdown on T-cell proliferation is like the inhibition observed after pU24-6A and pU24-6B transfection, this might suggest that pU24-6A/pU24-6B reduce Nedd4L expression with a consequent reduction of T-cell proliferation. Nedd4L controls the activity of TGF-β1, by triggering Smad2 and TGFBR1 ubiquitination, which in turn supports T-cell development, homeostasis, tolerance, and differentiation [72]. As a proof of concept, Smad2 phosphorylation and TGF-β1 secretion consequent to Nedd4L inhibition were significantly augmented in the cases of Nedd4L RNAi and pU24-6A and -6B treatment. These data suggest that pU24-6A and pU24-6B act like Nedd4L RNAi in reducing Nedd4L mRNA expression and enhancing TGF-β1 secretion via Smad2 phosphorylation.

Fyn tyrosine kinase is mainly localized in the oligodendrocyte plasma membrane [85]. It plays an important role in a range of signaling pathways during CNS development. Many studies have demonstrated that interactions between MBP and SH3-domain-containing proteins play a physiological role in oligodendrocytes and myelin formation, compaction, and overall stability [33,96]. A disruption in this MBP/Fyn-SH3 interaction can lead to myelin dysfunction and consequently MS. The data reported here suggest that U24-6B interactions with Fyn-SH3 are more like the interaction between MBP and Fyn-SH3 (i.e., both, broadly speaking, with μM affinities) than the interaction between U24-6A and Fyn-SH3 (which is in the mM range). Moreover, it was demonstrated that phosphorylation weakens the interaction, as is also the case between phospho-MBP and Fyn-SH3. The stronger interaction of U24-6B and Fyn-SH3 (relative to U24-6A) suggests that the introduction of a positive charge at Arg3 helps to stabilize binding. Indeed, some of the tightest binding ligands to Fyn, e.g., NS5A [97] and PI3-kinase peptide 2 (1A0N.pdb) [56,57], all have positive charges that flank the PxxP motif.

We previously observed that NK cells from MS patients have an increased susceptibility to HHV-6 infection, in particular in the presence of KIR2DL2 receptor [78]. For the first time, the interaction between KIR2DL2 and U24 was evaluated, as a possible trigger in NK cell activation control. We observed that KIR2DL2 positive NK cells were selectively inhibited by pU24-6A towards 721.221-ICP47-C1 cells, while MBP, pU24-6B and U24-7 did not affect the killing of these cells. In contrast, no killing was observed in 721.221-ICP47-A2 co-cultures with KIR2DL2 positive NK cells. These data showed a clear involvement of pU24-6A in NK cell activation, further supporting that HHV-6A may play a larger role in MS than HHV-6B.

Establishing a link between the viral protein U24 from HHV-6 and multiple sclerosis and more specifically, differentiating between association and causation, is complex, with many different hypotheses having been suggested. As discussed in [15], there is quite a bit of evidence associating HHV-6 to MS, but there is to date no proof indicating any clear causation. In addition, only a few proteins from HHV-6, besides U24, have been identified as possibly being implicated [15,21,55]. The data presented here, while not providing any definitive answers on association/causation, possibly provides some additional clues. For one, if U24’s role in MS was to mimic MBP because of their common PxxP motif, the data shown here would suggest that MS would have to be much more prevalent because the causative virus would have to be HHV-6B, which is present in ca. 95% of the population [15,98]. Alternatively, the data could suggest that a single PxxP motif may not be sufficient to mimic MBP well; indeed, LMP2A from Epstein–Barr virus (EBV), which has 4 PxxP motifs [13], has been associated with MS [99,100,101] and EBV is generally considered a more likely candidate as a viral risk factor for MS [11]. The findings that both pU24-6A and pU24-6B control T cell proliferation via Nedd4L inhibition and that pU24-6A’s most important role appears to be NK cell inhibition point to a possible importance of phosphorylation in the function of U24. Reduced proliferation of T cells has been identified as leading to susceptibility to multiple sclerosis [102]. Perhaps exposure to phosphorylated U24 from HHV-6A/6B increases this risk. Likewise, a number of studies have established a link between NK cells and MS, as reviewed extensively in [103]. The fact that pU24-6A specifically affects KIR2DL2 positive NK cells provides the first evidence that it might be this molecule that plays a role in the mechanisms triggering auto-immunity [78]. Finally, the data presented here provides additional support to previous reports in the literature for a biological role for U24 during MS: the ex vivo evaluation of the levels of U24 derived peptide IgGs showed the highest levels of IgG towards U24-6A and pU24-6A. Surprisingly, a higher number of subjects with high levels of IgG towards U24-6B, pU24-6B and U24-7 was found in MS patients in comparison with controls and neurolupus patients, confirming a higher immune response towards U24 and its phosphorylated form in MS patients.

## Figures and Tables

**Table 1:** Sequence alignment of myelin basic protein (MBP) and U24 from HHV-6A (U24-6A), -6B, and -7. The PxxP motif is indicated in dark gray, whereas the PY motif is shown in light gray. The subscript in the name of the peptide represents the residue numbers, with the numbering of MBP based on the human 18.5 kDa classic isoform. Negatively charged side-chains are shown in blue, while positively charged residues are in red. The phosphorylated peptides (pU24-6A and pU24-6B) are also included, with pT representing phospho-threonine. 15 residue peptides, with the sequences given here, were used in this study.

Name	Sequence															
MBP_93-107_	I	V	T	P	R	T	P	P	P	S	Q	G	K	G	R	
U24-6A_1-15_	M	D	P	P	R	**T**	P	P	P	S	Y	S	E	V	L	
pU24-6A_1-15_	M	D	P	P	R	**pT**	P	P	P	S	Y	S	E	V	L	
U24-6B_1-15_	M	D	R	P	R	**T**	P	P	P	S	Y	S	E	V	L	
pU24-6B_1-15_	M	D	R	P	R	**pT**	P	P	P	S	Y	S	E	V	L	
U24-7_1-15_	M	-	T	H	E	T	P	P	P	S	Y	N	D	V	M	L

**Table 2:** Parameters obtained from fitting the ITC data for binding of U24-6A, U24-6B and pU24-6B to hNedd4L-WW3* domains at 25 °C. Calculated values for Δ*G*° are also included. The parameters were obtained from fitting the data from three separate runs and averaging them. The errors represent ± one standard deviation. WW3* domain concentrations used in these experiments are in the caption of Figure 1.

	Target: hNedd4L-WW3*			
Ligand	*K_d_* (μM)	Δ*H*° (kJ/mol)	Δ*S*° (J/mol K)	Δ*G*° (kJ/mol)
U24-6A	8.5 ± 0.7	−59.5 ± 0.9	−103 ± 2	(−2.9 ± 0.1) × 10^1^
U24-6B	8.9 ± 0.9	−71 ± 4	−141.6 ± 0.9	(−2.9 ± 0.4) × 10^1^
pU24-6B	0.88 ± 0.01	−78.4 ± 0.6	−147 ± 2	(−3.46 ± 0.08) × 10^1^

## Data Availability

Data is available upon request to the corresponding author.

## Supplementary Materials

The following supporting information can be downloaded at: https://www.mdpi.com/article/10.3390/v14112384/s1, Figure S1: Sample HPLC trace for the purified U24-6B peptide. Figure S2: ITC and NMR titration results for U24-6A_ΔMD_ (U24-6A, residues 3 to 15, Table 1) and Fyn-SH3 domain. Figure S3: Structural models for the binding interaction between (a) U24 and (b) pU24 and hNedd4L-WW3. Table S1: Sequences and molecular weights (MW) of human Fyn-SH3 (Uniprot P06241; residue 82-142 underlined) and hNedd4L-WW3* (Uniprot Q96PU5; residues 497-530 underlined) used in this study. Table S2: Primer sequences and PCR conditions used to generate Nedd4L and the GAPDH control.
