# Supplementary material for: Studying the Interactions of U24 from HHV-6 in Order to Further Elucidate Its Potential Role in MS
Source: Viruses. 2022 Oct 28;14(11):2384. doi: 10.3390/v14112384 (PMC9696605; doi:10.3390/v14112384)
Supplement: Supplementary file 1 [file viruses-14-02384-s001.zip › viruses-1954468-supplementary.pdf]

viruses

MDPI

SUPPLEMENTARY INFORMATION to

Comment on nomenclature:

In this work, "U24" represents 24th protein coding open reading frame in the unique region of the HHV-6 genome (*u24*) and "pU24", its phosphorylated form (at Thr6). This nomenclature was chosen to be consistent with previous work in the literature and for simplicity, i.e., avoiding more complex terminology such as, e.g., pU24-6A-PT6 to indicate the phosphorylated protein (at Thr6), encoded by ORF *u24* from HHV-6A.

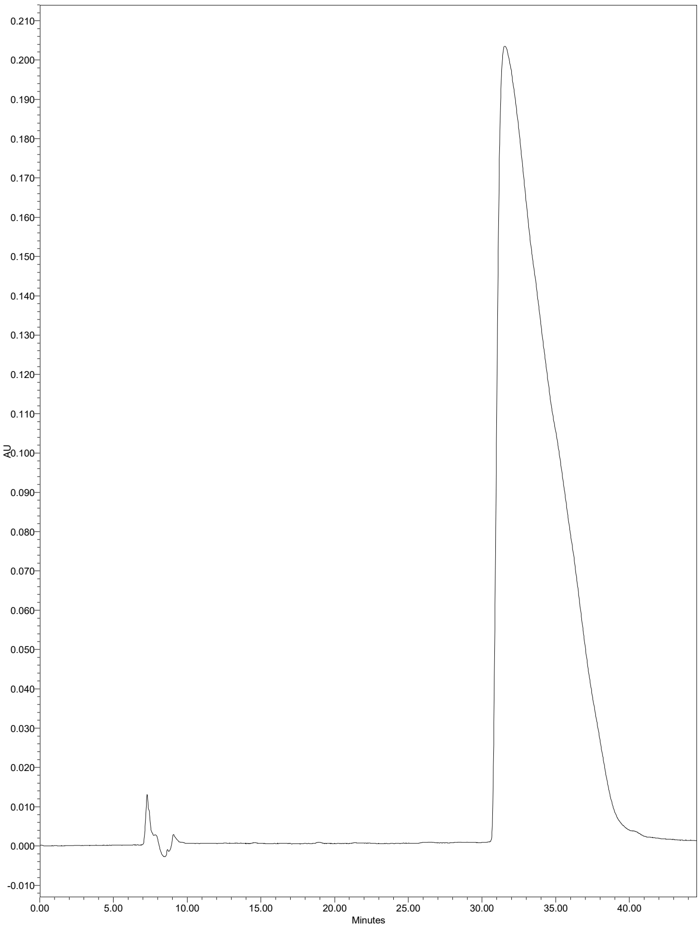

Figure S1. Sample HPLC trace for the purified U24-6B peptide, detected at a wavelength of 229 nm. As outlined in Materials and Methods, the purification process included multiple sequential HPLC runs.

Table S1. Sequences and molecular weights (MW) of human Fyn-SH3 (Uniprot P06241; residue 82-142 underlined below) and hNedd4L-WW3* (Uniprot Q96PU5; residues 497-530 underlined below) used in this study.

Name	Sequence	MW
Fyn-SH3	GSPGISGGGGGILDTGVTLFVALYDYEARTEDDLSFHKGEKFQILNSSEGDWWEARSLTTGETGYIPSNYVAPVD	8016.7
hNedd4L-WW3*	GSVTQSFLPPGWEMRIAPNGRPFFIDHNTKTTTWEDPRLKFPVHM	5241.0

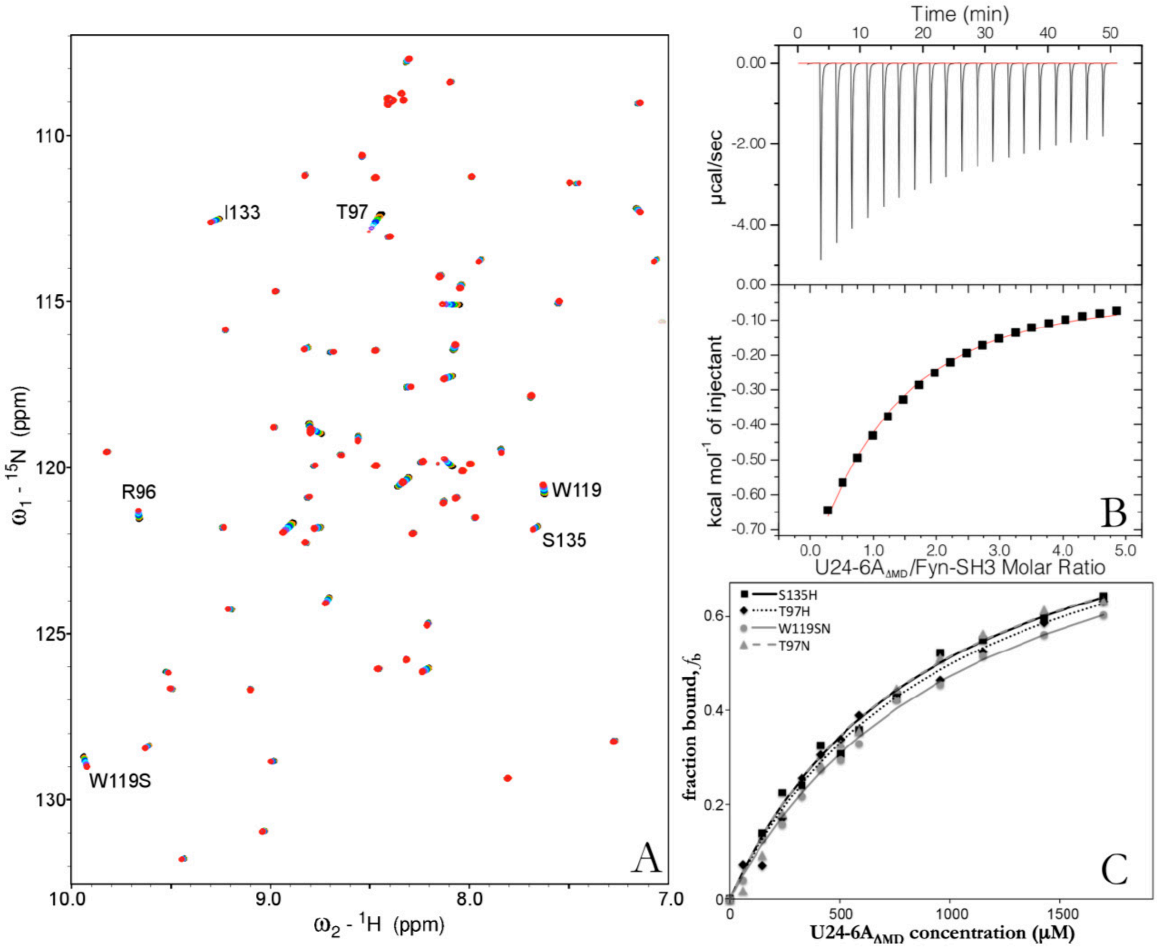

Figure S2. ITC and NMR titration results for U24-6A$_{\Delta\text{MD}}$ (U24-6A, residues 3 to 15, Table 1) and Fyn-SH3 domain. a) Overlay of ^{15}N-^{1}H HSQC spectra of ^{15}N-labelled Fyn-SH3 with U24 peptide added in a Fyn/U24 ratio of: 1:0 (black); 1:0.9 (orange); 1:2.0 (green); 1:3.1 (blue); 1:4.8 (cyan); 1:7.3 (purple) and 1:11 (red); b) ITC data for U24-6A$_{\Delta\text{MD}}$ peptide binding to Fyn-SH3 at 25 °C, containing (Upper) raw titration data and (Lower) integrated heat data (points) and best fit (red) to a "one set of sites" model. c) Fit of the proton chemical shift data for S135 (black, solid) and T97 (black, dotted); ^{15}N chemical data for W119S (grey, solid) and T97 (grey, dashed) from U24-6A$_{\Delta\text{MD}}$/Fyn-SH3 NMR titration.

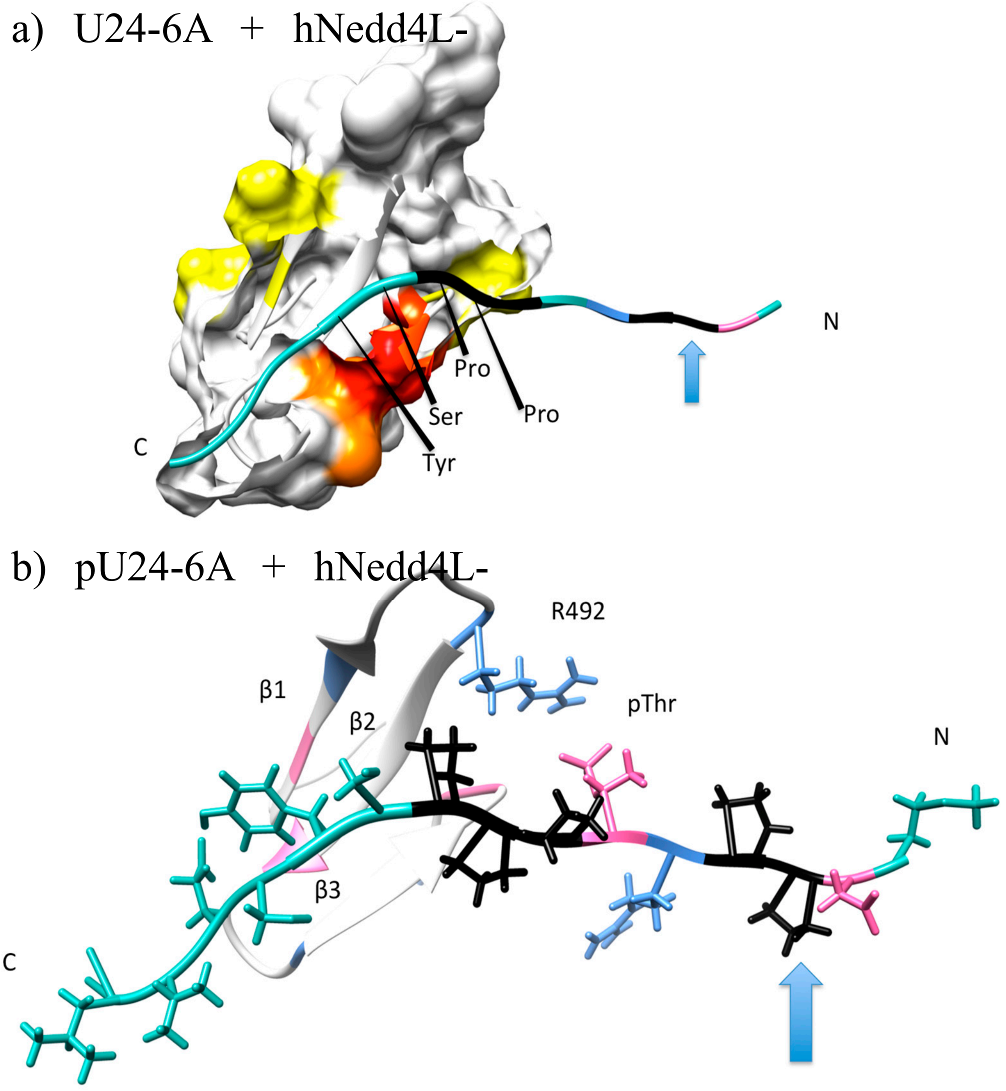

Figure S3. Structural models for the binding interaction between a) U24 and b) pU24 and hNedd4L-WW3*, as obtained from the predominant structure seen molecular dynamics simulations[1]. In a), the hNedd4L-WW3* domain is represented as a surface, whereas the U24-6A peptide is shown as a ribbon. In b), the hNedd4L-WW3* domain is represented as a white/blue/pink ribbon, whereas the pU24-6A peptide is shown as a multi-colored ribbon, with side-chains explicitly drawn in. Proline residues are shown in black, negatively charged residues in pink and positively charged residues in blue. The remaining residues are shown in cyan. The square ribbon illustrates the PxxP motif. The thicker rounded ribbon shows that PPSY region (i.e. PY motif), which is also labelled in a). Residues indicated in red-yellow are those found through NMR experiments to be perturbed (with red indicating the strongest chemical shift perturbation). The arrow indicates residue 3. This figure was generated using UCSF CHIMERA.

Table S2. The primer sequences and PCR conditions used to generate Nedd4L and the GAPDH control.

Gene	Forward Primers	Reverse Primers	Annealing Temperature
NEDD4L	5′-CTATGAATGGATTTGCCGAACT-3′	5′-GCAGAACTTGCTTGAAGAACAA-3′	60°C
GAPDH	5′-GAAGGTGAAGGTCGGAGTC-3′	5′-GAAGATGGTGATGGGATTTC-3′	57°C
